# Hospital and Patient Characteristics Regarding the Place of Death of Hospitalized Impending Death Patients: A Multilevel Analysis

**DOI:** 10.3390/ijerph16234609

**Published:** 2019-11-20

**Authors:** Shin-Ting Yeh, Yee-Yung Ng, Shiao-Chi Wu

**Affiliations:** 1 College of Nursing, National Taipei University of Nursing and Health Sciences, Taipei 11221, Taiwan; n2899@ms18.hinet.net; 2 Department of Medicine, School of Medicine, Fu Jen Catholic University Hospital, Fu Jen Catholic University, Taipei 24205, Taiwan; yyngscwu12@gmail.com; 3 Institute of Health & Welfare Policy, National Yang-Ming University, No.155, Sec.2, Linong Street, Taipei 11221, Taiwan

**Keywords:** dying, place of death, population register, multilevel analysis, hospital mortality

## Abstract

***Objectives:*** To explore the influence of hospital and patient characteristics on deaths at home among inpatients facing impending death. ***Method:*** In this historical cohort study, 95,626 inpatients facing impending death from 362 hospitals in 2011 were recruited. The dependent variable was the place of death. The independent variables were the characteristics of the hospitals and the patients. A two-level hierarchical generalized linear model was used. ***Results:*** In total, 41.06% of subjects died at home. The hospital characteristics contributed to 29.25% of the total variation of the place of death. Private hospitals (odds ratio [OR] = 1.32, 95% confidence interval [CI] = 1.00–1.75), patients >65 years old (OR = 1.48, 95% CI. = 1.42–1.54), married (OR = 3.15, 95% CI. = 2.93–3.40) or widowed (OR = 3.39, 95% CI. = 3.12–3.67), from near-poor households (OR = 5.16, 95% CI. = 4.57–5.84), having diabetes mellitus (OR = 1.79, 95% CI. = 1.65–1.94), and living in a subcounty (OR = 2.27, 95% CI. = 2.16–2.38) were all risk factors for a death at home. ***Conclusion:*** Both hospital and patient characteristics have an effect of deaths at home among inpatients facing impending death. The value of the inpatient mortality rate as a major index of hospital accreditation should be interpreted intrinsically with the rate of deaths at home.

## 1. Introduction

Because of the clinical competence provided by hospital personnel, dying in hospital is deemed preferable to dying at home for inpatients facing an impending death [1,2]. However, dying at home is considered psychologically more comfortable for patients facing an impending death because it gives family members and friends more time with the person and grants them more autonomy and privacy [3,4]. The choice of dying in a familiar environment such as the home is judged reasonable and might even be suggested by doctors [4]. 

The proportion of people dying at home ranges from 12% to 60% [5,6,7,8,9,10,11,12,13]. A study by Brazil et al. revealed that the rate of at-home deaths was 56% [14]. The rate of at-home deaths in Japanese patients was approximately 46% to 67% [15,16]. Among patients in Singapore, 29% died at home [17]. Tang et al. reported that the rate of at-home deaths in patients with cancer was approximately 32.4% to 43.6% [18,19]. Cohen et al. reported a strikingly large variation in the rate of home deaths (from 12% to 57%) in patients with cancer across 14 countries, namely Belgium, Canada, the Czech Republic, England, France, Hungary, Italy, Mexico, the Netherlands, New Zealand, South Korea, Spain, the United States, and Wales [20].

In addition to patients’ sex, age, education level, marriage status, income, and type of cancer [3,21,22,23,24,25,26,27,28,29], the accessibility and availability of health care services affect inpatients and their families in their choice between a hospital or an at-home death [21,25,29,30]. In Taiwan, the National Health Insurance (NHI) programs cover almost the entire population and reduce financial barriers to receiving medical care. Therefore, we investigated the effect of hospital and inpatient characteristics on the place of death under minimum influence from medical expenses. Whether hospitals play a role in the decision-making process of inpatients choosing an at-home death is of interest. If the lower inpatient mortality rate is due to inpatients who are facing impending death choosing an at-home death, the value of the inpatient mortality rate as a major index of hospital accreditation might be altered [6,31,32]. 

## 2. Methods

### 2.1. Study Cohort and Data Sources

The national register of deaths, health records of medical facilities, registry of beneficiaries, registry of contracted medical facilities, and inpatient expenditures from the National Health Informatics Project of the Ministry of Health and Welfare were linked using encrypted personal identification numbers and hospital IDs in this retrospective cohort study. 

### 2.2. Participants and Sampling

In 2011, 152,030 people (0.65% of the total population) died in Taiwan. After excluding people who died an accidental death, death by suicide or homicide, or before hospitalization, 97,203 people who had been hospitalized the day before their death were selected as inpatients facing impending death. Subsequently, 1577 deaths that occurred in psychiatric hospitals were excluded. Finally, 95,626 inpatients facing impending death from 362 hospitals were included for analysis in the present study. Because the NHI program covers most of the population, the use of national databases with encrypted personal IDs and death certificates prevented selection and participation bias [33].

### 2.3. Study Variables

The dependent variable selected was the place of death (either in hospital or at home). The independent variables included the characteristics of patients and hospitals. The patient characteristics included sex, age (<18, 18–39, 40–54, 55–64, 65–74, 75–84, ≧85), marital status (unmarried, married, divorced, widowed, missing), income (low-income households, near-poor households, moderate-income households, and high-income households), and cause of death (e.g., cancer, diabetes mellitus, heart disease, stroke, disease of the respiratory system, disease of the digestive system, and suicide). When reviewing the patients’ places of residence, the urbanization degree was categorized into the following five types: municipality, province, county, subcounty, and rural area. The hospital characteristics included the ownership status (public or private) and the accreditation status of the hospital (medical center, regional hospital, district teaching hospital, and district hospital). 

### 2.4. Statistical Analysis

All statistical analyses were performed using the SAS statistical (SAS system for Windows, version 9.3) and HLM 6.06 software packages. Numbers and percentages were used to describe the characteristics of the patients (Level 1) and the hospitals (Level 2). 

In the present study, inpatients from the same hospital were likely to be correlated [34]. Therefore, we applied two-level hierarchical generalized linear models (HGLMs) using the Bernoulli sampling method and logit link function to avoid the violation of the assumption of uncorrected errors [35], and to make the study result more robust [3]. At level 1, the characteristics of the patients were included. At level 2, the characteristics of the hospitals were included in this study. The model designed in the present study was of random intercept and fixed slope. We assumed that the effect of each patient’s factors was the same and the coefficient of each covariate was fixed across hospitals. This model design is a widely used approach in multilevel analyses [3,36]. 

In the HGLMs, the intraclass correlation coefficient (ICC) measured the proportion of total variance among hospitals [37]. In a normal hierarchical linear model, the estimation of the ICC requires both the random intercept (τ00) variance and the residual variance ($\sigma^{2}$): ICC = τ00/(τ00 + $\sigma^{2}$) [37]. However, if an HGLM presents no error term in the logit link function, it means there is no residual variance term ($\sigma^{2})$. Therefore, an approximate ICC was calculated assuming that the latent residual term followed a logistic distribution and using the variance of the logistic distribution π2/3 = 3.29. Under this model, the ICC was measured as τ00/[τ00 + (π2/3)] where π2/3 = 3.29 [38]. In addition, we calculated the $R^{2}$-type in different models. The $R^{2}$-type, which was used to represent the explanation of the model, was measured as [(VN − VF)/VN] × 100%. VN was the hospital-level variance of the null model, and VF was the variance of the full model [39].

The multilevel modeling followed a staged approach [40]. In the first stage, we used an unconditional model with no predictors to test for a significant between-hospital variability in the place of death. In the second stage, we included the estimations from several preliminary conditional models. Model 1 included the Level 1 predictors to determine if the effects of any of the Level 1 predictors varied across the study sample. Model 2 included the Level 2 predictors to determine if the effects of any of the Level 2 predictors varied across the study sample. Finally, we included all of the Level 1 and Level 2 predictors in Model 3.

### 2.5. Ethical Statement

This study was approved by the Institutional Review Board of National Yang-Ming University (approval number 99007) in Taiwan. All data sets were analyzed at the Health and Welfare Data Science Center (HWDC) because the results of the data analysis had to be verified by an examiner of the HWDC to ensure the protection of personal data.

## 3. Results

In this study, 60.84% of patients facing impending death were male, 30.19% were between 75 and 84 years old, 54.55% were married, 40.17% had a moderate income, 35.39% died of cancer, and 54.73% lived in a municipality. The patients were recruited at 67.84% from private hospitals and 32.16% from public hospitals. Specifically, 36.22%, 44.21%, 4%, and 15.57% were hospitalized in a medical center, regional hospital, district teaching hospital, and district hospital, respectively. In total, 41.06% (39,266 of 95,626) chose to die at home (Table 1).

In the bivariate analysis, female patients appeared more likely to choose to die at home than male patients (43.87% versus 39.25%, *p* < 0.001). The choice to die at home was significantly more common in elderly patients than in younger patients (respectively, 47.34%, 45.74%, and 39.77% in the age groups of 65–74 years old, 75–84 years old, and ≧85 years old versus 7.95%, 25.91%, 30.28%, and 38.98% in the age groups of <18 years old, 18–39 years old, 40–54 years old, and 55–64 years old, *p* < 0.001). The married and widowed patients were more likely to choose to die at home than the unmarried and divorced patients (respectively, 44.44% and 47.37% versus 14.01% and 17.85%, *p* < 0.001). Patients from moderate- to high-income households were also more likely to choose to die at home than patients from low-income and near-poor households (respectively, 60.48% and 38.35% versus 12.5% and 21.45%, *p* < 0.001). Patients with diabetes mellitus had a significantly higher rate of at-home deaths than patients with cancer, heart disease, stroke, and respiratory system disease (51.62% versus 41.55%, 38.94%, 44.78%, and 38.76%, respectively, *p* < 0.001). Inpatients living in municipalities were less likely to choose to die at home than those living in provinces, counties, subcounties, and rural areas (31% versus 35.38%, 40.32%, 60.08%, and 51.8%, respectively, *p* < 0.001). Patients in medical centers, regional hospitals, district teaching hospitals, and district hospitals chose to die at home in 39.24%, 45.4%, 37.65%, and 33.88% of cases, respectively. Compared with public hospitals, more inpatients hospitalized in private hospitals chose to die at home (31.50% versus 45.59%, *p* < 0.001) (Table 1).

In the HGLMs, the ICC measured the proportion of total variance that occurs among hospitals [37]. In the null model, the variation among hospitals was 1.36. The ICC was estimated to be 0.2925 [1.36/(1.36 + 3.29)]. This estimation indicated that the percentage of variation between hospitals was 29.25% of the total variation. The $R^{2}$-type in Model 1 was 25.00% [(1.36−1.02)/1.36×100%], indicating that patient characteristics could explain 25.00% of the variation in the place of death of inpatients facing impending death. The $R^{2}$-type in Model 2 was 5.15% [(1.36 − 1.29)/1.36 × 100%], indicating that hospital characteristics can explain 5.15% of the variation in the place of death of inpatients facing impending death. When both patient and hospital characteristics were entered in Model 3, the $R^{2}$-type was 30.15% [(1.36 − 0.95)/1.36 × 100%]. This result means that hospital and patient characteristics can explain 30.15% of the variation in the place of death of inpatients facing impending death (Table 2). Overall, patients >65 years old (odds ratio [OR] = 1.48, 95% confidence interval [CI] = 1.42–1.54), married (3.15, 2.93–3.40) or widowed (3.39, 3.12–3.67), from near-poor households (5.16, 4.57–5.84), having diabetes mellitus (1.79, 1.65–1.94), and living in a subcounty (2.27, 2.16–2.38) were more likely to be discharged from hospital after choosing to die at home compared with patients <65 years old, unmarried, with a high income, having other diseases (cancer, heart disease, and stroke), and living in a municipality. Compared with public hospitals, inpatients hospitalized in private hospitals were 32% more likely to be discharged from hospital after choosing to die at home (1.32, 1.00–1.75) (Table 2).

## 4. Discussion

The study demonstrated that the number of inpatients facing impending death who chose to die at home (41.06%) was lower than that in previous studies (57.7% in 2000 and 74.1% in 1971) [22,41]. This result might be related to the continuous urbanization and social transition in Taiwan. This phenomenon was evidenced by the lower proportion of inpatients living in municipalities choosing to die at home compared with those living in subcounties and rural areas (31% versus 60.08% and 51.8%, respectively, OR > 2) (Table 1 and Table 2). The limited living space in municipalities, which constrains the coffin-moving process in and out of apartments, might have led to patients and family members accepting an in-hospital death [3,24,41].

In the present study, older people, those who were married or widowed, or were from near-poor households, chose an at-home death, as in previous studies [22,26,30,41,42,43,44,45]. The fact that older patients tend to choose dying at home might be related to the traditional belief that ancestors will lead the deceased from home to the equivalent of paradise for Western monotheist religions. Dying at home does not only take the misfortune away, but it also brings good luck to the descendants [22]. Compared with unmarried and divorced patients, married or widowed patients are more often accompanied by family members [46]. Some studies have indicated that in-hospital deaths are associated with a lower quality and satisfaction, as well as complicated grief for the surviving family members [13,47,48]. Dying at home not only alleviates the loneliness of patients but also helps family members express their emotions, which in turn decreases the sorrow at the time of death [26]. 

The proportion of inpatients with diabetes mellitus facing impending death who chose an at-home death was significantly higher than that of patients with other diseases such as cancer, heart disease, or stroke (*p* < 0.001). This result might be related to their suffering and tiredness from the chronicity and comorbidities of diabetes such as nephropathy, neuropathy, and disability [23,27]. Therefore, patients with diabetes, and their families, tended to choose to die at home when the patients’ conditions deteriorated to the impending death stage.

A higher proportion of inpatients facing impending death in private hospitals chose to die at home, compared with public hospitals (1.51 times in Model 2 and 1.32 times in full model) (Table 2). This result indicates that inpatients are more likely to be discharged when they reach a critical condition at private hospitals compared with public hospitals [31,32]. The reason for private hospitals to discharge patients facing impending death might be related to their objective of low inpatient mortality rate, which advertises a better quality of care in terms of hospital accreditation. Although some researchers have considered the hospital standardized mortality ratio (HSMR) as a measure of health care quality, others have pointed out that considering HSMR as a measure of hospital quality leads to a possible skewing in the choice of the place of death. Our study evidenced that the more inpatients facing impending death there were who died at home, the lower the inpatient death rate. Therefore, combining inpatient and home death rates may yield a better index than inpatient death rate alone for the measurement of hospital health care quality.

According to Cohen’s definition [49], multilevel analysis is a more efficient method than regression analysis when the ICC is higher than 0.059. The ICC of 0.2925 in the null model in this study indicated a high degree of clustering in patients’ place of death between hospitals. The characteristics of hospitals and patients were associated with the place of death. Therefore, using a two-level (patients and hospitals) analysis to address the place of death was appropriate in this study. The characteristics of patients and hospitals explained 25.00% and 5.17% of the choice to die at home among patients facing impending death, respectively. This result indicates that patient characteristics matter more (4.85 times) than hospital characteristics in the choice to die at home. However, hospital characteristics also play a role in the choice to die at home. The effect of hospital characteristics cannot be ignored. 

This study had a limitation. Some parameters such as patient preference in place of death, functional status, and family support [50,51] were not included in our data set as other administration data sets did. Theoretically, the functional status of inpatients facing impending death might be similar. Furthermore, the marital status, family income, cause of death, and urbanization degree of the place of residence were entered into the model of this study for risk adjustment. Nevertheless, this study had two key strengths. First, inpatients from the same hospital were likely to be correlated. The two-level HGLMs used in this study solved the problem of inpatients’ nonindependence. This study not only improved the estimation of effects within patient units but also formulated and tested hypotheses on cross-level effects. In addition, the model partitioned the variance and covariance components among levels [52]. Second, this is the first study to explore the role of patient and hospital characteristics in the choice of death place among inpatients facing impending death. Apart from the patient factors, hospital characteristics played a role in the choice of an at-home death.

## 5. Conclusions

In total, 41.06% of inpatients facing impending death chose to die at home. The factors influencing the choice to die at home included hospitalization in a private hospital, >65 years old, married or widowed status, near-poor household, diabetes mellitus, and place of residence in a subcounty. The at-home death rate influences the inpatient mortality rate. Therefore, the value of the inpatient mortality rate as a major index of hospital accreditation should be interpreted intrinsically with the rate of deaths at home.

## Figures and Tables

**Table 1:** Situation of discharge for inpatients facing impending death in 2011: univariate analysis.

	Total	%	Death Place				*p* Value
			Hospital		Home		
			N	%	N	%	
**Total**	**95,626**	**100.00**	**56,360**	**58.94**	**39,266**	**41.06**	
**Patient’s characteristics**							
**Gender**							<0.001
Female	37,444	39.16	21,017	56.13	16,427	43.87	
Male	58,182	60.84	35,343	60.75	22,839	39.25	
**Age**							<0.001
<18	679	0.71	625	92.05	54	7.95	
18–39	2848	2.98	2110	74.09	738	25.91	
40–54	10,848	11.34	7563	69.72	3285	30.28	
55–64	13,318	13.93	8127	61.02	5191	38.98	
65–74	16,601	17.36	8742	52.66	7859	47.34	
75–84	28,867	30.19	15,663	54.26	13,204	45.74	
≧85	22,465	23.49	13,530	60.23	8935	39.77	
**Marriage**							<0.001
Unmarried	8409	8.79	7231	85.99	1178	14.01	
Married	52,168	54.55	28,982	55.56	23,186	44.44	
Divorce	5222	5.46	4290	82.15	932	17.85	
Widow	29,261	30.60	15,400	52.63	13,861	47.37	
missing	566	0.59	457	80.74	109	19.26	
**Income**							<0.001
Low-income households	3015	3.15	2638	87.50	377	12.50	
Near poor households	30,353	31.74	23,842	78.55	6511	21.45	
Moderate income	38,415	40.17	15,181	39.52	23,234	60.48	
High income	23,843	24.93	14,699	61.65	9144	38.35	
**Cause of death**							<0.001
Cancer	33,841	35.39	19,781	58.45	14,060	41.55	
Diabetes Mellitus	4287	4.48	2074	48.38	2213	51.62	
Heart diseases	7960	8.32	4860	61.06	3100	38.94	
Stroke	6932	7.25	3828	55.22	3104	44.78	
Diseases of the respiratory system	13,982	14.62	8562	61.24	5420	38.76	
Diseases of the digestive system	7530	7.87	4334	57.56	3196	42.44	
Suicide	447	0.47	291	65.10	156	34.90	
Others	20,647	21.59	12,630	61.17	8017	38.83	
**Urbanization degree**							<0.001
Municipality	52,336	54.73	36,110	69.00	16,226	31.00	
Province	4113	4.30	2658	64.62	1455	35.38	
County	8928	9.34	5328	59.68	3600	40.32	
subcounty	27,971	29.25	11,166	39.92	16,805	60.08	
Rural	2278	2.38	1098	48.20	1180	51.80	
**Hospital characteristics**							
**Ownership**							<0.001
Public	30,757	32.16	21,068	68.50	9689	31.50	
private	64,869	67.84	35,292	54.41	29,577	45.59	
**Accredited Hospital**							<0.001
Medical center	34,638	36.22	21,047	60.76	13,591	39.24	
Regional hospital	42,277	44.21	23,085	54.60	19,192	45.40	
District teaching hospital	3825	4.00	2385	62.35	1440	37.65	
District hospital	14,886	15.57	9843	66.12	5043	33.88	

**Table 2:** Factors affecting the discharge of inpatients facing impending death: two-level hierarchical generalized linear model.

	Two-Level Hierarchical Generalized Linear Model (N = 95,060) ^a^								
	Model 1			Model 2			Model 3		
	**Adj-OR**	**95% C.I.**	***p* Value**	**Adj-OR**	**95% C.I.**	***p* Value**	**Adj-OR**	**95% C.I.**	***p* Value**
Intercept	0.03	(0.02 − 0.03)	<0.001	0.43	(0.25 − 0.75)	<0.01	0.03	(0.02 − 0.05)	<0.001
**Patient’s characteristics**									
**Gender (Male:0)**									
Female	0.98	(0.95 − 1.02)					0.98	(0.95 − 1.02)	
**Age (<65:0)**									
≧65	1.47	(1.42 − 1.53)	<0.001				1.48	(1.42 − 1.54)	<0.001
**Marriage (Unmarried:0)**									
Married	3.15	(2.93 − 3.40)	<0.001				3.15	(2.93 − 3.40)	<0.001
Divorce	1.19	(1.08 − 1.32)	<0.01				1.19	(1.08 − 1.32)	
Widow	3.39	(3.13 − 3.67)	<0.001				3.39	(3.12 − 3.67)	<0.001
**Income (High income:0)**									
Low-income households	1.55	(1.37 − 1.76)	<0.001				1.55	(1.37 − 1.76)	<0.001
Near poor households	5.17	(4.57 − 5.84)	<0.001				5.16	(4.57 − 5.84)	<0.001
Moderate income	3.18	(2.81 − 3.60)	<0.001				3.17	(2.80 − 3.60)	<0.001
**Cause of death (Others:0)**									
Cancer	1.12	(1.07 − 1.17)	<0.001				1.11	(1.07 − 1.16)	<0.001
Diabetes Mellitus	1.79	(1.65 − 1.94)	<0.001				1.79	(1.65 − 1.94)	<0.001
Heart diseases	0.97	(0.91 − 1.02)					0.97	(0.91 − 1.03)	
Stroke	1.27	(1.18 − 1.35)	<0.001				1.27	(1.18 − 1.35)	<0.001
Diseases of the respiratory system	1.01	(0.96 − 1.06)					1.01	(0.96 − 1.06)	
Diseases of the digestive system	1.11	(1.04 − 1.19)	<0.01				1.11	(1.04 − 1.18)	<0.01
Suicide	0.62	(0.50 − 0.79)	<0.001				0.62	(0.50 − 0.79)	<0.001
**Urbanization degree (Municipality:0)**									
Province	1.35	(1.22 − 1.48)	<0.001				1.35	(1.22 − 1.48)	<0.001
County	1.55	(1.45 − 1.65)	<0.001				1.55	(1.45 − 1.66)	<0.001
subcounty	2.27	(2.16 − 2.38)	<0.001				2.27	(2.16 − 2.38)	<0.001
Rural	2.15	(1.91 − 2.41)	<0.001				2.16	(1.92 − 2.42)	<0.001
**Hospital characteristics**									
**Ownership (Public:0)**									
Private				1.50	(1.09 − 2.07)	<0.05	1.32	(1.00 − 1.75)	<0.05
**Accredited Hospital (Medical center:0)**									
Regional hospital				1.44	(0.81 − 2.55)		1.13	(0.69 − 1.86)	
District teaching hospital				0.77	(0.38 − 1.54)		0.68	(0.37 − 1.24)	
District hospital				0.77	(0.45 − 1.33)		0.66	(0.42 − 1.05)	
**Variance component of level 2 (τ_00_)**	**1.02**		**<0.001**	**1.29**		**<0.001**	**0.95**		**<0.001**
**R square ^b^ (%)**	**25.00**			**5.15**			**30.15**		

Note ^a^: We excluded the study sample whose marriage status was ‘missing’. Finally, there were 95,060 eligible samples for analysis in this study. The reference group of the dependent variable is people who are recorded dying in hospital. The variance component of the null model (τ00) is 1.36. Note ^b^: The R square was calculated by [(V_N_ − V_F_)/V_N_] × 100%, where V_N_ (1.36) was the variance component of level 2 in the null model and the V_F_ was the variance component of level 2 in the full model.

## References

[1] Cancernetwork.com Dying in Hospital May Be Preferable to Dying at Home. http://www.cancernetwork.com/articles/dying-hospital-may-be-preferable-dying-home.

[2] Higginson I.J., Sarmento V.P., Calanzani N., Benalia H., Gomes B. (2013). Dying at home—Is it better: A narrative appraisal of the state of the science. Palliat. Med..

[3] Gu D., Liu G., Vlosky D.A., Yi Z. (2007). Factors associated with place of death among the Chinese oldest old. J. Appl. Gerontol..

[4] WebMD Dying at Home: The Basics. http://www.webmd.com/healthy-aging/questions-answers-dying-at-home-medref.

[5] Gomes B., Calanzani N., Gysels M., Hall S., Higginson I.J. (2013). Heterogeneity and changes in preferences for dying at home: A systematic review. BMC Palliat. Care.

[6] Gruneir A., Mor V., Weitzen S., Truchil R., Teno J., Roy J. (2007). Where people die: A multilevel approach to understanding influences on site of death in America. Med. Care Res. Rev..

[7] Cohen J., Bilsen J., Addington-Hall J., Löfmark R., Miccinesi G., Kaasa S., Onwuteaka-Philipsen B., Deliens L. (2008). Population-based study of dying in hospital in six European countries. Palliat. Med..

[8] Cohen J., Houttekier D., Onwuteaka-Philipsen B., Miccinesi G., Addington-Hall J., Kaasa S., Bilsen J., Deliens L. (2010). Which patients with cancer die at home? A study of six European countries using death certificate data. J. Clin. Oncol..

[9] Currow D.C., Burns C.M., Abernethy A.P. (2008). Place of death for people with noncancer and cancer illness in South Australia: A population-based survey. J. Alliative Care.

[10] Murtagh F., Bausewein C., Petkova H., Sleeman K., Dodd R., Gysels M., Johnston B., Murray S., Banerjee S., Shipman C. (2012). Understanding Place of Death for Patients with Non Malignant Conditions: A Systematic Literature Review.

[11] Abel J., Pring A., Rich A., Malik T., Verne J. (2013). The impact of advance care planning of place of death, a hospice retrospective cohort study. BMJ Supportive Palliat. Care.

[12] Gomes B., Higginson I.J. (2008). Where people die (1974—2030): Past trends, future projections and implications for care. Palliat. Med..

[13] Mitchell S.L., Teno J.M., Miller S.C., Mor V. (2005). A national study of the location of death for older persons with dementia. J. Am. Geriatr. Soc..

[14] Brazil K., Howell D., Bedard M., Krueger P., Heidebrecht C. (2005). Preferences for place of care and place of death among informal caregivers of the terminally ill. Palliat. Med..

[15] Fukui S., Fukui N., Kawagoe H. (2004). Predictors of place of death for Japanese patients with advanced-stage malignant disease in home care settings: A nationwide survey. Cancer.

[16] Ikezaki S., Ikegami N. (2011). Predictors of dying at home for patients receiving nursing services in Japan: A retrospective study comparing cancer and non-cancer deaths. BMC Palliat. Care.

[17] Poulose J.V., Do Y.K., Neo P.S.H. (2013). Association between referral-to-death interval and location of death of patients referred to a hospital-based specialist palliative care service. J. Pain Symptom Manag..

[18] Tang S.T., Huang E.-W., Liu T.-W., Rau K.-M., Hung Y.-N., Wu S.-C. (2010). Propensity for home death among Taiwanese cancer decedents in 2001–2006, determined by services received at end of life. J. Pain Symptom Manag..

[19] Ko M.-C., Huang S.-J., Chen C.-C., Chang Y.-P., Lien H.-Y., Lin J.-Y., Woung L.-C., Chan S.-Y. (2017). Factors predicting a home death among home palliative care recipients. Medicine.

[20] Cohen J., Pivodic L., Miccinesi G., Onwuteaka-Philipsen B., Naylor W., Wilson D., Loucka M., Csikos A., Pardon K., Van den Block L. (2015). International study of the place of death of people with cancer: A population-level comparison of 14 countries across 4 continents using death certificate data. Br. J. Cancer.

[21] Beng A., Fong C.W., Shum E., Goh C.R., Goh K.T., Chew S.K. (2009). Where the elderly die: The influence of socio-demographic factors and cause of death on people dying at home. Ann. Acad. Med. Singap..

[22] Chiu M.K. (2006). Returning Home to Die? Changes in Death Locations and Related Factors, 1971–2000. NCCU J. Sociol..

[23] Kelley A.S., Ettner S.L., Wenger N.S., Sarkisian C.A. (2011). Determinants of Death in the Hospital Among Older Adults. J. Am. Geriatr. Soc..

[24] Lin H.C., Lin Y.J., Liu T.C., Chen C.S., Lin C.C. (2007). Urbanization and place of death for the elderly: A 10-year population-based study. Palliat. Med..

[25] Nakamura S., Kuzuya M., Funaki Y., Matsui W., Ishiguro N. (2010). Factors influencing death at home in terminally ill cancer patients. Geriatr. Gerontol. Int..

[26] Chu S.Y., Lin M.H., Chen T.J., Hwang S.J. (2008). Psychosocial and Clinical Factors Associated with Place of Death in Taiwan. Taipei City Med. J..

[27] Tang S.T., Liu T.W., Tsai C.M., Wang C.H., Chang G.C., Liu L.N. (2008). Patient awareness of prognosis, patient–family caregiver congruence on the preferred place of death, and caregiving burden of families contribute to the quality of life for terminally ill cancer patients in Taiwan. Psycho-Oncology.

[28] Taylor E.J., Ensor B., Stanley J. (2012). Place of death related to demographic factors for hospice patients in Wellington, Aotearoa New Zealand. Palliat. Med..

[29] Van Rensbergen G., Nawrot T.S., Van Hecke E., Nemery B. (2006). Where do the elderly die? The impact of nursing home utilisation on the place of death. Observations from a mortality cohort study in Flanders. BMC Public Health.

[30] Lin H.C., Lin C.C. (2007). A population-based study on the specific locations of cancer deaths in Taiwan, 1997–2003. Support Care Cancer.

[31] Lee T.F., Wu S.C. (2004). The Changes in Mortality Rate in Patients with Coronary Artery Bypass Surgery before and after the Implementation of a Casebased Prospective Payment System. Taiwan J. Public Health.

[32] Wu S.C., Chien L.N., Ng Y.Y., Chu H.F., Chen C.C. (2005). Association of Case Volume with Mortality of Chinese Patients after Coronary Artery Bypass Grafting Taiwan Experience. Circ. J..

[33] Hsing A.W., Ioannidis J.P. (2015). Nationwide population science: Lessons from the Taiwan National Health Insurance Research Database. JAMA Intern. Med..

[34] Chung W., Cho W.H., Yoon C.W. (2009). The influence of institutional characteristics on length of stay for psychiatric patients: A national database study in South Korea. Soc. Sci. Med..

[35] Robinson W.S. (2009). Ecological correlations and the behavior of individuals. Int. J. Epidemiol..

[36] Raudenbush S.W. (2004). HLM 6: Hierarchical Linear and Nonlinear Modeling.

[37] Parish S.L., Rose R.A., Andrews M.E., Shattuck P.T. (2009). Receipt of professional care coordination among families raising children with special health care needs: A multilevel analysis of state policy needs. Child. Youth Serv. Rev..

[38] Browne W.J., Subramanian S.V., Jones K., Goldstein H. (2005). Variance partitioning in multilevel logistic models that exhibit overdispersion. J. R. Stat. Soc. Ser. A (Stat. Soc.).

[39] Snidjers T., Bosker R.J. (1999). Multilevel Analysis: An Introduction to Basic and Advanced Multilevel Modeling.

[40] Ben-Zeev D., Ellington K., Swendsen J., Granholm E. (2011). Examining a cognitive model of persecutory ideation in the daily life of people with schizophrenia: A computerized experience sampling study. Schizophr. Bull..

[41] Lin M.H., Lu Y.S., Chen T.J., Hwang S.J. (2006). Trends in Place of Death of Cancer Patients in Taiwan. Taiwan J. Hosp. Palliat. Care.

[42] Burge F., Lawson B., Johnston G. (2003). Trends in the place of death of cancer patients, 1992–1997. Can. Med. Assoc. J..

[43] Costantini M., Balzi D., Garronec E., Orlandini C., Parodi S., Vercelli M., Bruzzi P. (2000). Geographical variations of place of death among Italian communities suggest an inappropriate hospital use in the terminal phase of cancer disease. Public Health.

[44] Gomes B., Higginson I.J. (2006). Factors influencing death at home in terminally ill patients with cancer: Systematic review. BMJ (Clin. Res. Ed.).

[45] Cabañero-Martínez M.J., Nolasco A., Melchor I., Fernández-Alcántara M., Cabrero-García J. (2019). Place of death and associated factors: A population-based study using death certificate data. Eur. J. Public Health.

[46] Turner V., Flemming K. (2019). Socioeconomic factors affecting access to preferred place of death: A qualitative evidence synthesis. Palliat. Med..

[47] Teno J.M., Clarridge B.R., Casey V., Welch L.C., Wetle T., Shield R., Mor V. (2004). Family perspectives on end-of-life care at the last place of care. JAMA.

[48] Wright A.A., Keating N.L., Balboni T.A., Matulonis U.A., Block S.D., Prigerson H.G. (2010). Place of death: Correlations with quality of life of patients with cancer and predictors of bereaved caregivers’ mental health. J. Clin. Oncol..

[49] Cohen J. (1988). Statistical Power Analysis for the Behavioral Sciences.

[50] Cohen J., Bilsen J., Miccinesi G., Lofmark R., Addington-Hall J., Kaasa S., Norup M., van der Wal G., Deliens L. (2007). Using death certificate data to study place of death in 9 European countries: Opportunities and weaknesses. BMC Public Health.

[51] Grundy E., Mayer D., Young H., Sloggett A. (2004). Living arrangements and place of death of older people with cancer in England and Wales: A record linkage study. Br. J. Cancer.

[52] Raudenbush S.W., Bryk A.S. (2002). Hierarchical Linear Models: Applications and Data Analysis Methods.

